# Hints on the Management of Labor

**Published:** 1877-11-20

**Authors:** A. H. Goelet

**Affiliations:** New York


### HINTS ON THE MANAGEMENT OF LABOR.

BY A. H. GOELET, M.D., OF NEW YORK.

The article "How to Manage a Case of Labor," by J. W. Martin, M. D., in the April number of your journal, page 86, has brought forth this article, and I intend it only as an appendix to that.

While I appreciate the excellence of his paper, I feel that there are other points necessary for the student and young practitioner, and these I propose to introduce here.

A great deal of time will be unnecessarily wasted if the advice of some authors on this subject be taken.

Meigs says, "Stay with a woman whose *os* is dilated to the size of a silver quarter;" and a great many follow his advice. But it is not always wise to do this. In primiparæ, sometimes the *os* is this size several days before the termination of labor, and even in some multiparæ it is this size twenty four hours bebefore. We all know women are apprehensive at this time, and the slightest thing will sometimes give them alarm. If a physician remains with a patient sooner than is necessary, and she should linger, she will be sure to imagine something is wrong; and this is but natural. She is ignorant of the exact degree of her advancement, but leaves this to her attendant. If she lingers long after he remains with her, she will lose confidence in his judgment, begin to fret and worry, and this will delay it only longer.

If, upon being called to a case of labor, he should find the edges of the *os* thick, rounded, moist, and yielding in a woman who has borne children, he should remain with her if the *os* be considerably dilated, and the pains are severe and frequent; but if the *os* be only slightly dilated, and the pains weak and infrequent, he may either leave her till the pains increase, or he may give m xx. or m xxx. fluid extract of ergot, and remain with her, repeating the dose in half an hour if necessary. (My experience has been that it often takes hour for ergot to act well after it is given. Authorities say twenty minutes.)

If the *os* is found as above described in a woman who has never borne children, there is less risk in leaving her; and, if it be decided to give ergot, it should be given more cautiously and in smaller doses. If, on the other hand, he should find the edges of the *os* thin, sharp, hard, resisting, and undilatable, in any case, the wisest plan is to give m x. or m xii. of Majendie's Solution of Morphine hypodermically, or by the mouth, if there be no objection; and if there be any idiosyncrasy against opium, give a full dose of atropia in some way; then he may leave her until the *os* is relaxed; but first he must gain her confidence by a positive assurance that it is entirely unnecessary for him to remain with her, and that he will return in a few hours. This, while it may or may not produce sleep, acts happily on the *os* by relaxing it, and does not diminish the contractions of the uterus. In a few hours the *os* will be found soft and relaxed.

If rupture of the perineum be imminent, a slight incision with the thumb lancet on each side of the raphæ will avoid a rupture down the center, which is attended with more danger. After delivery, the slight incisions made will be found to amount to nothing, and will soon heal.

[This suggestion to puncture the perineum we think very questionable—ED.]

With regard to the delivery of the placenta, the sooner you can make the patient comfortable the better. Therefore as soon as you have cut the cord, which you may do as soon as respiration is well established, make traction upon the cord with one hand, and with the other grasp the uterus through the abdominal walls, and, as it were, squeeze out the placenta.

It is entirely unnecessary to remain an hour after the delivery of the placenta, as advised by certain authorities. As soon as the uterus can be felt firmly contracted through the abdominal walls, and all clots have been removed, she may be left with perfect impunity, if she be directed to retain the recumbent position. But should there be apprehension of hemorrhage, give half drachm fld. ext. ergot before leaving her.

